# Cortical thickness gradients in structural hierarchies

**DOI:** 10.1016/j.neuroimage.2015.02.036

**Published:** 2015-05-01

**Authors:** Konrad Wagstyl, Lisa Ronan, Ian M. Goodyer, Paul C. Fletcher

**Affiliations:** a Brain Mapping Unit, Department of Psychiatry, University of Cambridge, Cambridge, CB2 3EB, UK; b Developmental and Life-course Research Group, Department of Psychiatry, University of Cambridge, Cambridge, CB2 8AD, UK; c Cambridge and Peterborough Foundation Trust, UK

## Abstract

MRI, enabling *in vivo* analysis of cortical morphology, offers a powerful tool in the assessment of brain development and pathology. One of the most ubiquitous measures used—the thickness of the cortex—shows abnormalities in a number of diseases and conditions, but the functional and biological correlates of such alterations are unclear.

If the functional connotations of structural MRI measures are to be understood, we must strive to clarify the relationship between measures such as cortical thickness and their cytoarchitectural determinants. We therefore sought to determine whether patterns of cortical thickness mirror a key motif of the cortex, specifically its structural hierarchical organisation. We delineated three sensory hierarchies (visual, somatosensory and auditory) in two species—macaque and human—and explored whether cortical thickness was correlated with specific cytoarchitectural characteristics. Importantly, we controlled for cortical folding which impacts upon thickness and may obscure regional differences.

Our results suggest that an easily measurable macroscopic brain parameter, namely, cortical thickness, is systematically related to cytoarchitecture and to the structural hierarchical organisation of the cortex. We argue that the measurement of cortical thickness gradients may become an important way to develop our understanding of brain structure–function relationships. The identification of alterations in such gradients may complement the observation of regionally localised cortical thickness changes in our understanding of normal development and neuropsychiatric illnesses.

## Highlights

• Cortical thickness mirrors organisation of sensory hierarchies and cytoarchitecture.
• Patterns replicated in macaque and humans.
• Hierarchical level is strongly predicted by a gradient of cortical thickness.
• Thickness gradient matches functional hierarchy in human visual cortex.

## Graphical abstract

**Figure:**
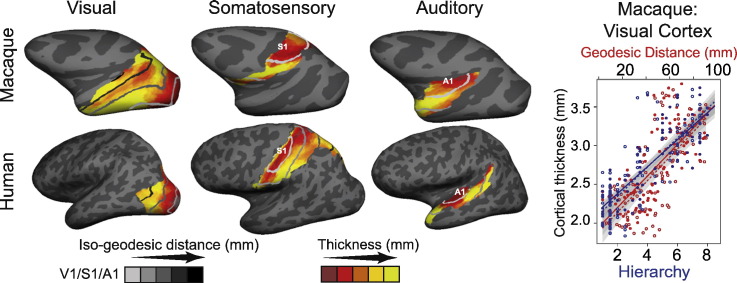

## Introduction

Although elegant work has established an indisputable general relationship between cortical morphology, cytoarchitecture and function (Broca, 1861; Fischl et al., 2008), the precise nature of this relationship is unclear. Moreover, there is a considerable degree of inter-individual variability in the large-scale structural features of the brain, which adds further ambiguity to regional analysis of both structure and function (Rajkowska and Goldman-Rakic, 1995; Amunts et al., 2007). The goal of the current study was to determine whether one widely used morphological measure—cortical thickness—varies across the cortex in a manner predicted by other cytoarchitectural and functional characteristics. Specifically, we used existing knowledge about the cellular, laminar and hodological patterns within the cortex to determine whether cortical thickness measures followed a corresponding pattern. Such an observation would lend weight to the proposition that macroscopic structural measures obtained using MRI, act as a useful marker of underlying neurophysiology. If true, then individual differences in cortical thickness measures would offer a possible interpretational framework for the neural bases of psychiatric disorders.

Central to our investigation is the principle that the brain processes information through pathways known as functional hierarchies. Computational hierarchical models describe a system of functionally specialised regions with feedforward and feedback connections (Rao and Ballard, 1999). Feedforward connections communicate incoming sensory information, while feedback connections relay experience-derived predictions (Bastos et al., 2012), which alter the response to the incoming signal (Bullier et al., 1996). Each cortical region processes particular features of the incoming sensory stimulus. These features tend to become increasingly specific and complex on ascending the hierarchy (Bond, 2004). For example, the primary visual cortex, V1, is responsive to basic image features present in most stimuli (Hubel and Wiesel, 1959), whereas area MT is preferentially responsive to certain types of motion (Tootell et al., 1995) and the fusiform face area (FFA) is selective to faces (Kanwisher et al., 1997), suggesting MT and FFA are higher than V1 in the visual processing hierarchy (Grill-Spector and Malach, 2004). Similar patterns of increasing stimulus selectivity and complexity are found in auditory (Wessinger et al., 2001; Okada et al., 2010) and somatosensory (Iwamura, 1998) hierarchies.

The cortex also demonstrates hierarchical organisation of regions based on structural characteristics, such as cortico-cortical connectivity. These data are most commonly derived through histological tracer studies in which dyes are injected to follow efferent axons to their target areas (Pandya and Sanides, 1973). The relative positions of two cortical areas within in a hierarchy are determined by the ratio of connection types, which can be feedforward (generally from lower to higher regions) or feedback (higher to lower). Feedforward axons tend to project from more superficial cortical layers while feedback axons project from deeper layers (Pandya and Sanides, 1973; Markov et al., 2013). Tracer studies have revealed connection-based hierarchies in visual, somatosensory, motor (Felleman and Van Essen, 1991), auditory (Hackett et al., 1998) and prefrontal regions (Barbas, 1986).

At a microscopic scale, the cytoarchitecture of cortical areas mirrors their structural hierarchical organisation. The degree of laminar differentiation is, for example, strongly related to a region's connectivity and position within a sensory structural hierarchy (Barbas, 1986). Primary sensory regions have six clearly defined cytoarchitectural layers whereas, for higher-level areas, the laminar differentiation is less distinct. More generally, it has been observed that primary sensory regions have increased neuronal density compared to other cortical areas (Collins et al., 2010; Scholtens et al., 2014).

While laminar structure and density might reflect the relative position of a region in a structural hierarchy (Scholtens et al., 2014), these patterns cannot yet be quantified using MRI. This limits the degree to which human structural and functional processing can be correlated *in vivo*. However, it may be that other more accessible parameters such as cortical thickness are related to cortical cytoarchitecture and can be used as an effective surrogate marker of laminar differentiation and, by extension, structural hierarchy. For example, neuronal density and cortical thickness are generally inversely correlated (la Fougère et al., 2011; Cahalane et al., 2012), and both exhibit a similar rostral-caudal gradient across the cortex. These results tentatively support the hypothesis that a gradient of cortical thickness, from thinner primary sensory areas to thicker higher-order areas, may be a useful biomarker of changes in cortical cytoarchitecture, structure and potentially functions of the hierarchy.

While there is some debate over the precise ordering of individual regions (Hilgetag et al., 1996), structural hierarchies do show consistent similarities to functional hierarchies. For example, in the visual cortex, area MT is consistently higher than areas V1 and V2 (Felleman and Van Essen, 1991; Hilgetag et al., 1996; Markov et al., 2013; Grill-Spector and Malach, 2004); in the auditory cortex, there is a matching progression from the auditory core, to the belt regions, and then to the superior temporal gyrus and superior temporal sulcus (Hackett et al., 1998; Wessinger et al., 2001; Okada et al., 2010); and in the somatosensory cortex, the hierarchy progresses caudally across the post-central gyrus, through BA (Brodmann area) 3a, 3b, 1, 2 and 5 (Felleman and Van Essen, 1991; Iwamura, 1998). Structural and functional hierarchies may therefore describe the same principle of cortical organisation. However, several electrophysiological studies have demonstrated higher-order characteristics in lower order regions (Hegde and Felleman, 2007; Lee et al., 2002), suggesting that sensory processing is not so easily simplified. Nevertheless, if structural hierarchies index functional hierarchies, then inter-individual variability in cortical cytoarchitecture and thickness may reflect differences in hierarchical function.

However, empirical evidence that cortical thickness is a marker of structural hierarchy is lacking. A relationship between the thickness of a cortical area and its relative hierarchical position may be obscured by the mechanics of cortical gyrification, which cause gyri to be on average 20% thicker than sulci (Van Essen and Maunsell, 1980; Fischl and Dale, 2000). However, these effects, although relatively under-explored, can easily be identified and accounted for using MR-based surface reconstruction approaches (Fig. 3).

**Fig. 1:**
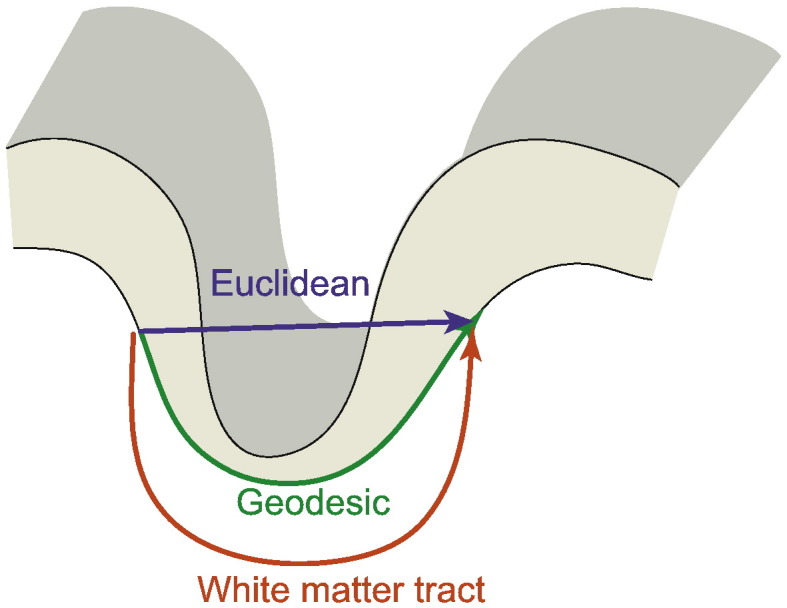
Distance measures. Geodesic distance measures the shortest path between two points across the white matter (or pial) surface of the cortex. Euclidean distance is the shortest distance through 3-dimensional space. White matter tract distance approximates the length of an axon connecting two regions.

In summary, this study began by evaluating whether cortical thickness is a surrogate marker of laminar differentiation. Subsequently, we investigated whether connectivity and functional hierarchies are organised in accordance with structural gradients. We used structural MR images from macaque and human subjects. For each subject, thickness gradients were quantified for three separate hierarchies in each species, namely, visual, somatosensory and auditory. Hierarchical level was identified in the macaque using standardised parcellation maps and tracer-derived hierarchies (Felleman and Van Essen, 1991; Barbas, 1986). With respect to the human data set, given a lack of precise knowledge about the layout of structural hierarchies, we used geodesic distance from the primary sensory region as a proxy measure of hierarchical position, having validated this approach against macaque hierarchies (Fig. 1). Geodesic distance also offers a means of analysis which obviates differences between the various hierarchical models that exist. Finally, we compared thickness against a functionally derived visual hierarchy. Importantly, all measures of cortical thickness were adjusted to account for the local effects of folding.

## Methods

### MRI acquisition

Structural MRI data from one macaque (*Macacca mulatta*) and 83 human subjects were analysed. The macaque data were acquired using a 3 T Acheiva Phillips Scanner at Boston University School of Medicine. A T1-weighted 3DMPRAGE image (TR = 7.09, TE = 6, Flip = 8) was obtained through the brain with 0.6 x 0.6 x 0.6 mm voxel size. The human subjects were scanned according to the Human Connectome Project (Van Essen et al., 2013; Milchenko and Marcus, 2013) protocol, using 3 T Siemens Skyra Connectome Scanner at Washington University, St. Louis. A T1-weighted 3DMPRAGE image (TR = 2400, TE = 2.14, TI = 1000, Flip = 8, FOV = 224 × 224) was obtained through the brain with 0.7 x 0.7 x 0.7 mm voxel size.

### FreeSurfer reconstruction and cortical thickness

Cortical reconstructions were created using FreeSurfer software (Dale et al., 1999; Fischl et al., 1999a,b). Cortical thickness was measured as the shortest distance between each vertex on the white matter surface and the pial surface (Fischl and Dale, 2000).

### Parcellation and cortical hierarchies

The macaque surface reconstruction was registered to the F99 atlas using FreeSurfer & Caret software (Van Essen et al., 2001, 2012a; Van Essen, 2002). The F99 atlas contains several cortical parcellation schemes including FVE91 (Felleman and Van Essen, 1991), used to outline somatosensory and visual cortical areas, PHT00 (Paxinos et al., 2000) used for auditory areas and M132 (Markov et al., 2013) atlas used for comparison with laminar differentiation data.

Macaque visual and somatosensory hierarchies were taken from Felleman and Van Essen (Felleman and Van Essen, 1991); regions were delineated in the FVE91 parcellation scheme. Successive levels in the hierarchy were given numerical values, such that V1 has a hierarchical level of 1, V2 is level 2, etc. Several areas in the original visual hierarchy were omitted from this analysis: 7b, ER, 36, HC, FEF, 46, TF and TH. The first 4 of these are non-visual regions, TF and TH part of the parahippocampal cortex, which is structurally different to normal neocortex. Areas FEF and 46 are frontal regions and not part of the hypothesised continuous structural gradient. The somatosensory hierarchy was simplified from the somatomotor hierarchy (for discussion of motor hierarchy, see Limitations section).

The macaque auditory hierarchy is described by several studies (Kaas et al., 1999; Rauschecker and Scott, 2009; Bond, 2004; Barbas, 1986), which outline hierarchies with consistent characteristics, including anterolateral progression. For this study, we took the hierarchy from Barbas (1986), a cytoarchitecture and tracer-defined hierarchy with 6 levels. Architectonic regions in the hierarchy are well described and each level is an aggregate of several areas in the PHT00 parcellation scheme. In this study, the auditory hierarchy was manually outlined into these 6 cortical regions.

Human reconstructions were registered to the PALS-B12 atlas in Caret (Van Essen et al., 2012b) and into its component gyri and sulci in FreeSurfer (Destrieux et al., 2010).

The human visual cortex was parcellated with a matching functional hierarchy, adapted from Grill-Spector and Malach (2004) using regions found in the PALS-B12 atlas and V1 as identified by FreeSurfer (Hinds et al., 2008). Primary somatosensory cortex (BA 3b) (parcellated in FreeSurfer; Fischl et al., 2008), post-central gyrus and sulcus, and superior parietal gyrus (Iwamura, 1998) were included as somatosensory regions. Primary auditory cortex (Heschel's gyrus/transverse temporal gyrus), superior temporal gyrus and transverse temporal sulcus were included as auditory regions (Rauschecker and Scott, 2009).

Due to an absence of matching functional hierarchy and parcellation schemes, geodesic distance from the primary sensory regions was used as a surrogate marker for hierarchical level for somatosensory and auditory hierarchies.

### Geodesic distance

There is some debate over the precise details of cortical hierarchies (Hilgetag et al., 1996; Bond, 2004; Barbas, 1986; Felleman and Van Essen, 1991), but there is evidence that, within the visual hierarchy, hierarchical level increases with distance from the primary sensory region (Grill-Spector and Malach, 2004; Markov et al., 2013). For this reason, we used geodesic distance from the primary sensory region to each randomly parcellated target region as a surrogate measure for hierarchical level. Geodesic distance was measured as the shortest path across the white matter surface between the vertices closest to the centre of each region (Fig. 3). Distance was first validated against hierarchical level and cortical thickness in the all three macaque sensory hierarchies and human functional visual hierarchy, before being implemented as the sole measure of hierarchical level in human auditory and somatosensory hierarchies. We acknowledge that the use of the geodesic distance as a marker for hierarchical level was necessarily speculative in human somatosensory and auditory cortex but, in the absence of an empirically validated means of identifying levels, we adopted these measures as a reasonable surrogate.

### Individual variability and folding

**Fig. 2:**
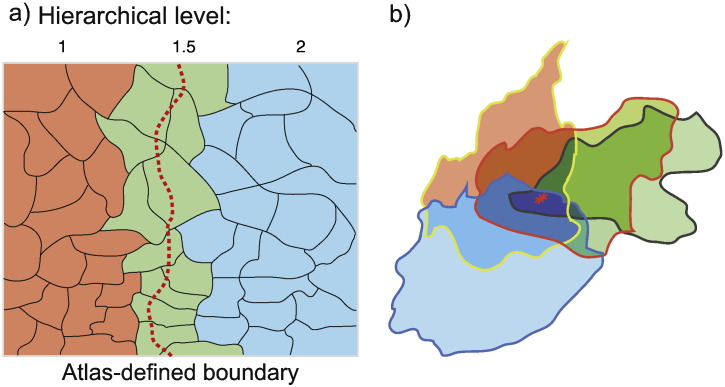
Boundaries and parcellation. (a) Addressing individual variability in atlas-defined boundaries. The dashed red line represents an atlas boundary between the orange area with hierarchical level 1 and the blue area with hierarchical level two. Randomly parcellated regions crossed by the red line are given the mean of the hierarchical levels. (b) The random parcellation process is repeated 10 times, averaging cortical thickness values across parcellations to mitigate gyral–sulcal thickness differences (Fig. 3).

**Fig. 3:**
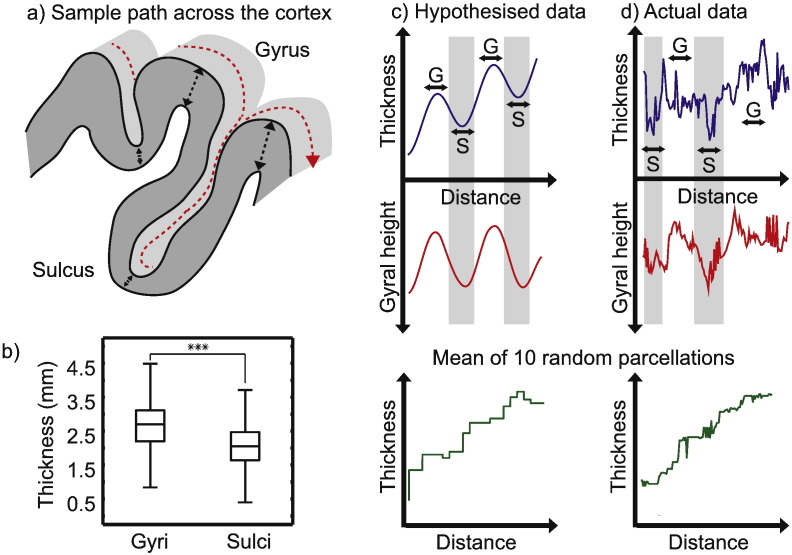
The effect of folding on cortical thickness. (a) The red line shows a sample path across the cortical surface. Gyri are visibly thicker than their adjacent sulci. (b) Unsmoothed MRI thickness values across one cortical hemisphere. Gyri are significantly thicker than sulci in a two-sample *T*-test (*p* < 0.001). (c) Hypothesised effect of folding on thickness values obscuring gradient. (d) Actual data taken from a sample path proceeding anteriorly from V1 in one subject. Averaging across 10 random surface parcellations mitigates the effect of gyral–sulcal position.

Automated parcellation schemes are to some extent limited by individual variability. For example, there is a two-fold intersubject variability in the surface area of V1 (Andrews et al., 1997), and many borders are not consistently associated with large-scale morphological landmarks (Welker, 1990; Amunts et al., 2007). To address the uncertainty over precise border locations, the cortex was randomly parcellated into 100 regions of approximately equal surface area; any region containing a border between different hierarchical levels was assigned the average value of the levels (Fig. 2a). By repeating random parcellation 10 times and averaging the thicknesses (Fig. 2b), we minimise the effect of folding (Fig. 3) and the bias of each individual random parcellation scheme.

### Thickness validation

Because high cortical myelin content, as found in the primary sensory regions, can lead to underestimations in some MR measurements of cortical thickness (Glasser and Van Essen, 2011), we validated FreeSurfer cortical thickness in the human auditory cortex by comparing it to previously published histological data (Triarhou, 2007; von Economo and Koskinas, 1925). Average FreeSurfer values for human auditory cortex were 2.84 mm and 2.88 mm for the left and right hemispheres (SD = 0.31 mm), while histological measurement gives an estimate of 2.9 mm. The close match with histological data is in keeping previous studies, which have validated FreeSurfer thickness measurements with histological comparison (Fischl and Dale, 2000; Cardinale et al., 2014).

### Macaque histological data

Measurements of laminar differentiation in the macaque cortex were derived from an aggregate of multiple histological studies (Barbas, 1986; Barbas and Rempel-Clower, 1997; Dombrowski et al., 2001). Brain areas in the M132 atlas were ranked according to their cytoarchitectonic differentiation, taking into account features such as neuronal density and granularisation of layer 4. For example, the primary visual cortex with strong laminar differentiation has laminar differentiation type 8, while areas with less clearly defined laminar structure have progressively lower rankings.

### Statistics

Statistical analysis was carried out using Matlab & R (MATLAB, 2010; R Core Team, 2013). All relationships were tested using Spearman's partial rank correlation controlling for the effect of hemisphere. We also tested macaque data using a linear model, with individual hemispheres as covariates and human data were tested with a linear mixed-effects model, with individual hemispheres as covariates and individual subjects included as a random effect.

## Results

### Macaque cortical thickness and cytoarchitecture

**Fig. 4:**
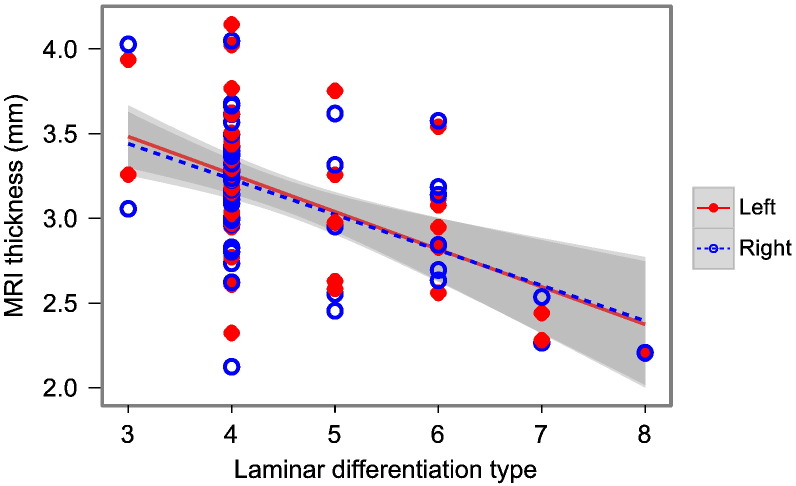
Macaque cortical thickness and cytoarchitecture. MRI thickness—corrected for folding—against laminar differentiation type (*r*_s_ = − 0.39, *p* < 0.001). Laminar differentiation type is a cytoarchitectural ranking scale, with 6-layered primary sensory cortex having type 8, while less well differentiated cortical regions are given progressively lower rankings (Barbas, 1986; Barbas and Rempel-Clower, 1997; Dombrowski et al., 2001).

Regional laminar differentiation data were compared with folding-corrected, MRI-derived cortical thickness values using Spearman's partial rank correlation controlling for the effect of hemisphere. In agreement with our hypothesis, cortical thickness correlated negatively with laminar differentiation type (*r*_s_ = − 0.39, *p* < 0.001)(Fig. 4).

### Cortical thickness, hierarchical level and geodesic distance

**Fig. 5:**
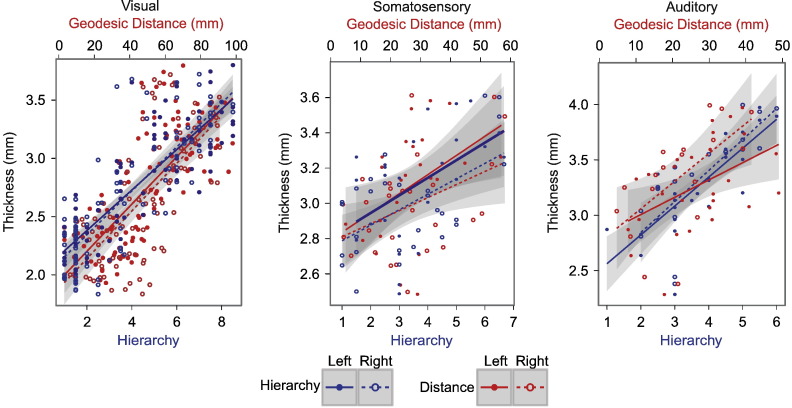
Macaque structural hierarchies. Graphs showing that cortical thickness correlates with structural hierarchy and geodesic distance from the primary sensory area in the macaque (see Table 1 for statistical results). Blue lines and points show that as hierarchical level increases cortical thickness (mm) also increases, for visual, somatosensory and auditory hierarchies. Red lines and points show geodesic distance (mm)—the putative surrogate of hierarchical level—increasing with cortical thickness for all three sensory hierarchies. Solid lines and filled circles show left hemisphere, dashed lines and hollow circles show right hemisphere.

Macaque data were analysed using Spearman's partial rank correlation and a linear model, controlling for the effect of hemisphere (see Table 1). In agreement with our hypothesis, hierarchical level was significantly correlated with and predicted by cortical thickness in visual, somatosensory and auditory cortices, where hierarchical level increased with thickness (Figs. 5, 8 & 9). In the somatosensory cortex, the correlation was not as strong but was still significant.

Hierarchical level was significantly correlated with and predicted by geodesic distance in all three hierarchies. This result is important for human somatosensory and auditory hierarchies, where there was no available parcellation for the literature-based hierarchies.

Finally, geodesic distance from the primary sensory region was also predicted by cortical thickness in the visual, somatosensory and auditory cortices. Again the relationship in the somatosensory cortex was not as strong, but the correlation was still significant.

**Fig. 6:**
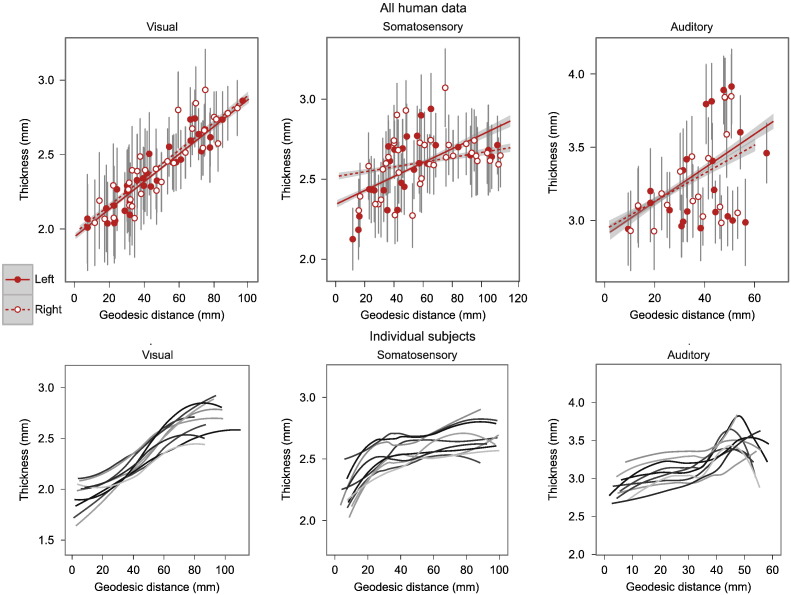
Human structural hierarchies. Graphs show that cortical thickness increases with geodesic distance from the primary sensory area in human sensory cortices (see Table 1 for statistical results). Geodesic distance is used as a surrogate marker of structural hierarchical level. Upper graphs: data from all human subjects. Points represent mean thickness value for a random sample region across 83 subjects; error bars represent population standard deviation. Lines show linear models with grey 95% confidence band for population trend. Solid lines and filled circles show left hemisphere, dashed lines and hollow circles show right hemisphere. Lower graphs: trend lines of individual data from 10 subjects. These plots show the consistent structural gradients across individuals and also a degree inter-individual structural variability. This variability may be of interest in healthy development and psychopathology.

Human data were analysed using Spearman's partial rank correlation and a linear mixed-effects model, controlling for the effect of hemisphere and the random effects of individuals (see Table 1). In agreement with the macaque data and our original hypothesis geodesic distance was significantly predicted by cortical thickness in the visual, somatosensory and auditory cortices (Figs. 6, 8 and 9). For somatosensory and auditory cortices the correlations were not as strong but were still significant (Fig. 9).

**Fig. 7:**
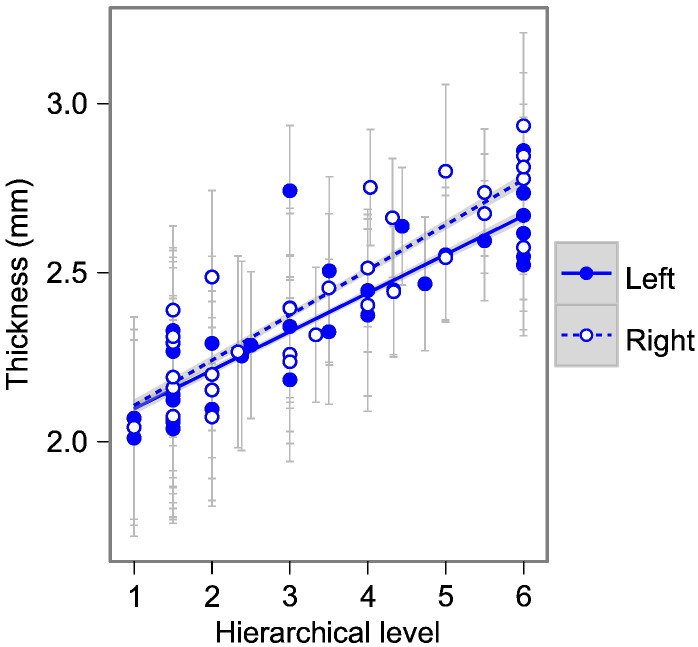
Human functional hierarchy. Cortical thickness (mm) increases with fMRI-derived functional hierarchical level (Grill-Spector and Malach, 2004) in humans (See Table 1 for statistical results). Points represent mean thickness value for a random sample region across 83 subjects; error bars represent population standard deviation. Lines show linear model with grey 95% confidence band for population trend. Solid lines and filled circles show left hemisphere, dashed lines and hollow circles show right hemisphere.

**Fig. 8:**
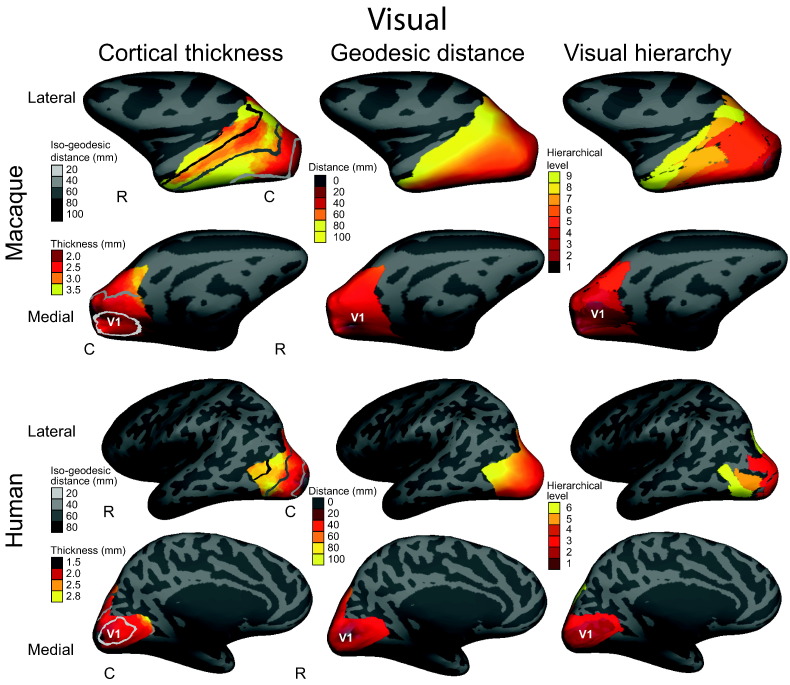
Visual cortex: cortical thickness, geodesic distance and hierarchical level for a single macaque and human. Left column: folding-corrected cortical thickness (mm) for the visual cortex with greyscale lines of iso-geodesic distance (mm) from the primary visual cortex (V1). Middle column: continuous measure of geodesic distance from V1. Right column: structural hierarchical level of visual regions based on axonal tracer studies in the macaque (Felleman and Van Essen, 1991) and functional hierarchical level of visual regions based on fMRI in the humans (Grill-Spector and Malach, 2004). Correlations between cortical thickness, geodesic distance and hierarchical level are highly significant (*p* < 0.001). Data overlaid on inflated left hemispheres, lateral and medial views. Rostral (R), caudal (C).

**Fig. 9:**
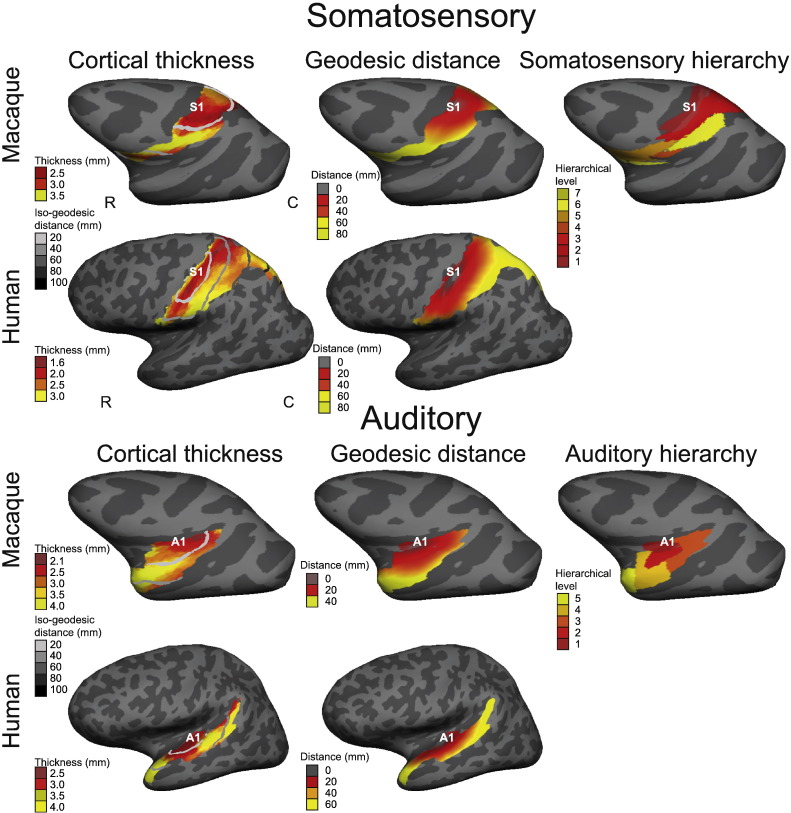
Somatosensory and auditory cortices: cortical thickness, geodesic distance and hierarchical level for a single macaque and human. Left column: folding-corrected cortical thickness (mm) with greyscale lines of iso-geodesic distance (mm) from the primary sensory cortex (S1 or A1). Middle column: continuous measure of geodesic distance from S1/A1. Right column: structural hierarchical level of somatosensory and auditory regions based on axonal tracer studies and cytoarchitecture in the macaque (Felleman and Van Essen, 1991; Barbas, 1986). Matching hierarchies and cortical parcellations were not available for humans. Correlations between cortical thickness, geodesic distance and hierarchical level are highly significant. Data overlaid on inflated left hemispheres, lateral views. Rostral (R), caudal (C).

For the visual cortex, functional hierarchical level was significantly predicted by cortical thickness and geodesic distance from the primary visual cortex (Figs. 7 & 8).

## Discussion

In this investigation, we tested the hypothesis that cortical thickness may be a useful marker of cytoarchitecture—one that can be used to identify gradients in sensory structural hierarchies. Our results demonstrate a strong relationship between cortical thickness, laminar differentiation and estimated hierarchical position for visual, auditory and somatosensory hierarchies. Furthermore, our data are compatible with a close coordination of cortical structure and function. Critically, the findings overall suggest that patterns of cortical thickness, which are readily measurable in vivo with structural neuroimaging, can be interpreted meaningfully in terms of underlying patterns that relate directly to cortical function. They offer a framework for analysing and interpreting cortical thickness measures in health and disease.

### Interpreting cortical thickness

In the macaque, cortical thickness—corrected for effect of folding—was negatively correlated with laminar differentiation. While previous studies have shown a relationship between thickness and neuronal density (la Fougère et al., 2011; Collins et al., 2010; Cahalane et al., 2012), an inverse correlation with laminar differentiation has not previously been demonstrated. Moreover, throughout the cortex, there are a number of inter-dependent and predictable relationships between various cytoarchitectural properties. For example, neuronal density can be reliably related to intracortical connectivity, such that lower neuronal density is associated with increased dendritic arborisation and density of synapses (Schüz and Palm, 1989; Elston, 2003; Cullen et al., 2010). The inter-relationship between neuronal density, laminar differentiation and cortical thickness may be useful in considering thickness changes in studies of structural MRI.

### Cortical thickness, structural hierarchies and function

Cortical hierarchies have previously been characterised by laminar differentiation and by layer-specific cortico-cortical connections. Here we have shown that cortical thickness may be a further indicator of hierarchical level. Each of these measures brings its own advantages and functional implications.

First, layer-specific interregional connections are considered functionally as indexing feedforward (signals going from lower to higher regions) and feedback connections (higher to lower signalling). Hierarchical connectivity patterns such as these have previously been characterised using axonal tracer studies indicating that (i) feedforward connections predominantly originate in upper cortical layers and terminate in the lower layers of a target region and (ii) feedback connections project from lower and terminating in upper layers (Pandya and Sanides, 1973; Markov et al., 2013).

Interregional feedforward and feedback connectivity has been related to integration of sensory information in the context of increasingly influential predictive coding models (Bastos et al., 2012). Within such models, the balance of feedforward/feedback connections determines functional organisation. Thus, the patterns of laminar-specific connectivity have important implications for how structure may govern and shape function.

A second measure of structural hierarchy is cytoarchitectural. The pattern of feedforward/feedback innervation is closely linked to laminar differentiation (Barbas, 1986; Barbas and Rempel-Clower, 1997), neuronal density and even dendritic tree size and spine density (Scholtens et al., 2014). Furthermore, some of the changes in neuronal density are layer specific, where caudal cortical regions have increased density in supragranular layers (Charvet et al., 2013), which give rise to feedforward axons (Pandya and Sanides, 1973; Markov et al., 2013). Rather than describing an identical, repeated neuronal microcircuit, these measures emphasise systematic variation in intracortical connectivity throughout the hierarchies.

Our present study shows that cortical thickness follows a similar gradient to hierarchies of connectivity and cytoarchitecture and might therefore offer a third related marker of structural hierarchy. Critically, unlike laminar connectivity and cytoarchitectural patters, thickness gradients are accessible to standard neuroimaging tools. Thus, the identification of this non-invasive, in vivo marker of cytoarchitectural trends offers a framework for interpreting cortical thickness patterns which are frequently reported, although poorly understood, in health and disease. Moreover, our observation of a gradient of cortical thickness within the fMRI-based human visual hierarchy (Grill-Spector and Malach, 2004) suggests a hitherto unestablished mapping between structural and functional hierarchies. Although such a relationship remains speculative, our results support the idea that structural regularities have a direct functional correlate.

### Cortical hierarchies in development and disease

Laminar connectivity, cytoarchitecture and thickness change over the course of development. There is little change in the number of feedforward connections postnatally (Batardière et al., 2002), while both feedback connections, synaptic density and cortical thickness increase postnatally up to a peak and then decrease (Barone et al., 1995; Goldman-Rakic, 1987; Price et al., 2006; Shaw et al., 2008). Based on this rationale, we hypothesise developmental changes in thickness gradients mark changes cortical cytoarchitecture and connectivity that support higher-level sensory processing.

During childhood, thickness increases at a differential rate across the cortex, with various regions achieving peak thickness at different time points between the ages of 7 and 11 (Shaw et al., 2008; Gogtay et al., 2004). Of note, hierarchical gradients develop in a gradual and non-uniform way, with primary sensory areas reaching peak thickness before secondary and higher association areas (Gogtay et al., 2004). It has been observed that the timing of thickness peaks is correlated with functional development. For example, in the visual system, the occipital cortex peaks at roughly the same age that children develop letter acuity and global motion detection (Lewis and Maurer, 2005). These observations suggest that by quantifying the thickness gradient in a sensory structural hierarchy, it may be possible to more accurately interpret the stages of development.

More generally, a gradient-based approach to structural analysis obviates another important limitation of such analysis, namely, the high inter-individual variation in the pattern of morphology and cytoarchitecture across the cortex (Rajkowska and Goldman-Rakic, 1995). By assessing gradient differences across structural hierarchies as opposed to individual regions, the ambiguity of regional identity is somewhat mitigated. Moreover, larger-scale regional analyses might be informative in conditions of atypical neurodevelopment such as autism and schizophrenia, which are associated with complex distributed changes across the cortex and are not reducible to a single morphological or functional abnormality (Chung et al., 2005; Ecker et al., 2013; Kuperberg et al., 2003; Fletcher and Frith, 2009; Rimol et al., 2012; Ronan et al., 2012).

### Cortical thickness and folding

It is important to note that observations of the relationships between morphology, cytoarchitecture and functional organisation were made having taken steps to account for the effect of folding, which causes gyri to be significantly thicker than sulci (Fischl and Dale, 2000) (Fig. 3b). This folding effect may obscure the large-scale thickness gradient of interest, and we therefore minimised its effect through the random parcellation-based smoothing (Figs. 2, 3c and d). In fact, this approach is almost equivalent to surface-based Gaussian kernel smoothing.

It is noteworthy that, in general, individual differences in cortical folding patterns could obscure or introduce group cortical thickness differences. Smoothing at a study-appropriate scale may help to mitigate these folding effects. Indeed this might underpin increases in sensitivity to group thickness differences observed when applying surface-based Gaussian smoothing kernels at 30 mm FWHM (Lerch and Evans, 2005).

### Limitations

A number of limitations attend this study. First, there is an ongoing debate over the precise organisation of structural hierarchies and it can be difficult to assert that any given sub-region belongs to a single specific level within a hierarchy (Hilgetag et al., 1996; Markov et al., 2013). Moreover, it is simplistic to suppose that structural hierarchies comprehensively and exhaustively describe functional organisation and information flow, and there are several possible measures of hierarchical function (Bond, 2004). We aimed to mitigate these concerns by demonstrating only the general trend of cortical thickness in structural hierarchies. Having done this in those hierarchies about which we were most confident, a complementary analysis using geodesic distance as a surrogate marker of hierarchical levels was used to further validate the approach. Although this approach overlooks some of the subtleties of structural hierarchies, we nevertheless believe our results persuasively demonstrate a variability in cortical thickness that closely correlates with previously established markers of structural hierarchy and certain measures of functional hierarchy.

In this study, we also confirmed the validity of our measure of cortical thickness, given the confounding effect of cortical myelin content (see Methods). High cortical myelin content, as found in the primary sensory regions, can lead to underestimations in some MR measurements of cortical thickness (Glasser and Van Essen, 2011). However, our analysis of the auditory cortical thickness indicated a close match between *FreeSurfer* cortical thickness measurement and histological data, which is in keeping previous validation studies (Fischl and Dale, 2000; Cardinale et al., 2014).

Some of our results in the somatosensory and auditory cortices, while significant, are not particularly strong (*r*_s_ < 0.35). This may in part be due to the hierarchies being relatively small, both in terms of the number of hierarchical levels and the area over which they extend. Nevertheless, the results remain significant suggesting that these structural principles do still apply in these sensory areas.

We note that this analysis was confined to the cortical thickness gradients in sensory processing hierarchies, such that these results may not be generalisable to motor, frontal and prefrontal cortices. While there is evidence for the existence of motor and frontal hierarchies, both structural (Felleman and Van Essen, 1991; Barbas, 1986; Goulas et al., 2012) and functional (Badre, 2008), several considerations prevent the simple extension of the hypothesis to include these pathways for this experiment. First, the structure of frontal and prefrontal cortices is far more variable (Dombrowski et al., 2001). Unlike sensory cortices, primary motor and premotor areas have an agranular cytoarchitecture and are particularly thick (Brodmann, 1909), despite having clear laminar differentiation (Barbas, 1986). Similarly, the frontal cortex does not follow the same inverse relationship between neuronal density and cortical thickness seen in all other cortical regions (la Fougère et al., 2011). Moreover, the direction of information flow is reversed, with signals predominantly travelling caudally from frontal ‘abstract’ areas towards the primary motor cortex (Badre and D'Esposito, 2009). Therefore, while cortical thickness gradients may be of interest in frontal-motor hierarchies, they are unlikely to follow the pattern seen in sensory cortices.

Finally, it should be noted that this experiment used data from a single macaque as a proof-of-concept analysis, capitalising on the high degree of specificity with which the functional organisation is known in this species. Although this necessarily limits the generalisability of this analysis, the closely matched findings in the 83 human subjects support the hypothesis that gradients of cortical thickness co-vary with hierarchical level and suggest that this may be a general principle of brain organisation and structure.

## Conclusions

Our findings offer a new framework for the interpreting cortical thickness measured using structural MRI. Cortical thickness gradients are significantly correlated with structural hierarchical level of sensory processing hierarchies in macaque and human subjects. Multiple lines of evidence suggest that this relationship is further characterised by predictable changes in cortical cytoarchitecture and connectivity. The results also suggest a close coupling between cortical structure and functional demand, such that higher-order visual areas tend to be thicker. We propose that analysis of these gradients may advance our understanding of the structure–function relationship in normal and atypical neurodevelopment.

## Figures and Tables

**Table 1:** Results of statistical analyses. Partial rank correlations and linear/linear mixed-effects models from macaque and human data.

	Spearman's partial rank correlations				Linear and mixed-effects models					
	Cortical thickness and hierarchy				Cortical thickness predicts hierarchy					
	Macaque		Human		Macaque			Human		
	*r*_s_	*p*	*r*_s_	*p*	β	*t*	*p*	*β*	*t*	*p*
Visual	0.73	< 0.001	0.62	< 0.001	3.9	21.1	< 0.001	4.9	90.7	< 0.001
Somatosensory	0.35	< 0.001			3.1	4.5	< 0.001			
Auditory	0.82	< 0.001			2.3	8.1	< 0.001			

	Geodesic distance and hierarchy				Geodesic distance predicts hierarchy					

	*r*_s_	*p*	*r*_s_	*p*	*β*	*t*	*p*	*β*	*t*	*p*

Visual	0.90	< 0.001	0.90	< 0.001	0.09	27.3	< 0.001	0.07	151.8	< 0.001
Somatosensory	0.74	< 0.001			0.09	8.9	< 0.001			
Auditory	0.74	< 0.001			0.08	6.9	< 0.001			

	Cortical thickness and geodesic distance				Cortical thickness predicts geodesic distance					

	*r*_s_	*p*	*r*_s_	*p*	*β*	*t*	*p*	*β*	*t*	*p*

Visual	0.65	< 0.001	0.63	< 0.001	31	13.8	< 0.001	66.3	91.7	< 0.001
Somatosensory	0.30	< 0.001	0.31	< 0.001	21.1	3.5	0.001	48.8	25.2	< 0.001
Auditory	0.55	< 0.001	0.34	< 0.001	13.1	4	< 0.001	18.6	19.1	< 0.001
